# FKBP5 isoforms shape immune pathways related to tumor tolerance

**DOI:** 10.1038/s41420-026-03047-5

**Published:** 2026-03-26

**Authors:** Simona Romano, Laura Marrone, Gennaro Acanfora, Valeria Di Giacomo, Marialuisa Alessandra Vecchione, Elena Vigliar, Antonino Iaccarino, Nicola Russo, Emanuele Sasso, Guendalina Froechlich, Massimiliano Cacace, Chiara Malasomma, Rosetta Abate, Simona Caggiano, Federica Aracri, Yichuan Xiao, Giancarlo Troncone, Tommaso Russo, Maria Fiammetta Romano

**Affiliations:** 1 Department of Molecular Medicine and Medical Biotechnology, Federico II University, Naples, Italy; 2 Department of Public Health, Federico II University, Naples, Italy; 3 Animal Models Core Facility Biogem s.c.a.r.l, Ariano Irpino (AV), Italy; 4 CEINGE-Biotecnologie Avanzate Franco Salvatore s.c.a.r.l., Naples, Italy; 5 ImGen-T, S.r.l., Naples, Italy; 6 Centro Servizi Veterinari, Federico II University, Naples, Italy; 7 CAS Key Laboratory of Tissue Microenvironment and Tumor, Shanghai Institute of Nutrition and Health, University of Chinese Academy of Sciences, Shanghai, China

**Keywords:** Tumour immunology, Tumour immunology

## Abstract

FKBP51 is a multifunctional immunophilin that regulates key cellular pathways, including NF-κB signaling, AKT activation, and steroid receptor trafficking. Alternative splicing of the FKBP5 gene generates a shorter isoform, FKBP51s, lacking the C-terminal TPR domain mediating protein‒protein interactions. Previous studies support the hypothesis that splicing of the FKBP5 gene underpins the mechanisms crucial for establishing control over T lymphocyte expansion and that the balance between the canonical and spliced isoforms could be pivotal in the immune system’s capacity to finely tune the intensity and span of the immune response.

To investigate the impact of FKBP51 splicing, we generated a knock-in mouse model (hu*FKBP5*) expressing only the full-length human FKBP51 isoform, thereby ablating all splice variants. While the heterozygous animals were viable and fertile, the homozygous hu*FKBP5*^+/+^ mice presented a sub-Mendelian frequency, infertility, and widespread lymphoid infiltrates, suggesting impaired immune homeostasis. In a syngeneic melanoma model, hu*FKBP5*^+/-^ mice presented a potent antitumor response characterized by reduced tumor growth, increased lymphocyte infiltration, elevated levels of cytotoxic markers (perforin, Bax, cleaved caspase-3 and -7, gasderminE), and the upregulation of CCR7 and CXCR5 on tumor-infiltrating lymphocytes. By employing synthetic mRNAs, we demonstrated that FKBP51 retains functional equivalence across species in driving lymphocyte activation. The FKBP51s isoform functions as a dominant-negative regulator of murine lymphocyte effector functions. These findings support the conclusion that inhibiting FKBP51 splicing preserves lymphocyte effector activity and prevents their transition toward resting or exhausted states. In the tumor context, this translates into a heightened antitumor immune response and a reduction in tumor tolerance.

## Introduction

FK506 binding protein 51 (FKBP51) is a 51 kDa m.w. member of the family of proteins that bind the immunosuppressant drug FK506 [1]. The *FKBP5* human gene is located on chromosome 6 and consists of 11 exons. FKBP51 possesses two N-terminal FK domains (FK1 and FK2), with only FK1 showing peptidyl-prolyl cis/trans isomerase activity (PPIase) and binding to FK506, while FK2 is inactive as a PPIase but seems to retain the ability to bind the drug and contains an ATP/GTP-binding sequence [1]. The C-terminal region of FKBP51 harbors three TPR (tetratricopeptide repeat) domains responsible for protein-protein interactions [1]. In addition to the canonical *FKBP5* mRNA consisting of all 11 exons, alternative splicing generates a truncated isoform that lacks the last 4 exons and thus encodes a short protein (FKBP51s) that is devoid of the TPR domain and carries a different C-terminus due to a frameshift [1, 2].

FKBP51 is involved in numerous biological processes, including (i) the trafficking of steroid receptor heterocomplexes through binding to heat shock protein 90 [3]; (ii) the enhancement of NF-κB activation through direct interaction with IKK subunits and the promotion of the assembly and enzymatic activity of the IKK complex [4]; and (iii) the activation of Akt/AKT1 through TPR binding to Akt, promoting K63 ubiquitination of Akt [5]. This process is supported by the FK-mediated interaction with PHLPP, which stabilizes the E3-ubiquitin ligase TRAF6 [5].

Increasing evidence support immunoregulatory roles for FKBP51 [6–8]. This immunophilin has been widely implicated in pro-inflammatory NF-κB signaling, as demonstrated by attenuated microglial activation in *Fkbp5*-deficient mice [6]. Kästle et al. investigated FKBP51 modulation of inflammation in a murine house‑dust mite-induced pulmonary model [7]. In this context, they confirmed that FKBP51 interacts with IKK complex members and promotes NF‑κB signaling by facilitating the nuclear translocation of p50/p65 dimers, leading to enhanced expression of inflammatory mediators. FKBP51 silencing reduced p50/p65 nuclear localization and attenuated cytokine and chemokine release, supporting a role for FKBP51 in controlling inflammatory responses in steroid‑insensitive settings [7]. A recent study reports that pharmacological inhibition of FKBP51 influences immune cell polarization and alleviates autoimmune diseases, reinforcing immunoregulatory roles for FKBP51 [8].

Considering that some of the functions of FKBP51 depend on the TPR domain [2–4], the functional role of FKBP51s is of particular interest. By lacking the C-terminal TPR domain, FKBP51s acts as a dominant-negative isoform of canonical FKBP51 [4, 5]. Furthermore, available evidence suggests that FKBP51s displays isoform-specific, TPR-independent functions, most notably acting as a cochaperone for PD-L1 [9, 10].

We were the first that identified FKBP51s in peripheral blood mononuclear cells (PBMCs) from melanoma patients, where its expression was driven by coinhibitory immune receptor signaling through the PD-L1/PD-1 pathway [2]. Our studies show that FKBP51s exerts a dominant negative effect on full-lenght FKBP51 capacity to activate NF-κB [4] and Akt [5], thereby counterbalancing the pro-inflammatory aspects of the canonical isoform. Moreover, we identified FKBP51s as a PD-L1 cochaperone that facilitates the post-translational maturation of PD-L1, promoting its glycosylation and stable expression at the plasma membrane [9]. FKBP51s/PD-L1 interaction not only stabilizes PD-L1 but also enhances immune evasion by the tumor, contributing to cancer immune escape [9]. Building on our original identification of FKBP51s as a PD-L1 cochaperone, we demonstrated that FKBP51s regulates PD-L1 expression in glioblastoma and contributes to tumor resistance mechanisms, validating the relevance of FKBP51 alternative splicing in immune checkpoint regulation [10]. In a glioma model, Yamaguchi et al. reported that the high expression of PD-L1 was decreased by celecoxib due to post-transcriptional regulation of *FKBP5* gene [11].

A recent investigation by our group revealed dynamic regulation of FKBP51 isoforms during immune activation [12]. Upon lymphocyte stimulation, both isoforms respond to T-cell receptor activation, yet they display distinctly opposite trends [12]. The upregulation of canonical FKBP51 closely parallels the presence of proliferative markers, while the induction of FKBP51s occurs strategically during two key phases of lymphocyte activation: during the development of regulatory T cells (Tregs) and at the conclusion of the proliferative burst as the cells transit back to a resting state [12].

Based on these observations, we hypothesize that the two isoforms of the *FKBP5* gene significantly underpin the mechanisms essential for establishing control over T lymphocyte expansion and that the balance between the canonical and short isoforms could be pivotal in the immune system’s capacity to finely tune the intensity and span of the immune response, maintaining homeostasis within the immune landscape. To address this hypothesis, we generated a knock-in mouse model in which the canonical human FKBP51 cDNA was inserted into exon 2 of the murine locus, followed by a strong polyadenylation signal. This genetic configuration ensures the exclusive expression of the full-length human isoform and prevents the generation of alternative splice variants. In the homozygous state, this model was expected to completely lack all isoforms derived from alternative splicing.

While full replacement of the murine gene with the human *FKBP5* gene [13, 14] does not result in a discernible phenotype, our model reveals that the selective ablation of putative isoforms derived from alternative splicing in the homozygous state profoundly impairs mouse development and fertility. Upon the implantation of a syngeneic melanoma tumor, the heterozygous animals elicit a vigorous immune response resulting in remarkable suppression of tumor growth.

## Results

### Features of the humanized FKBP5 mice

**Fig. 1:**
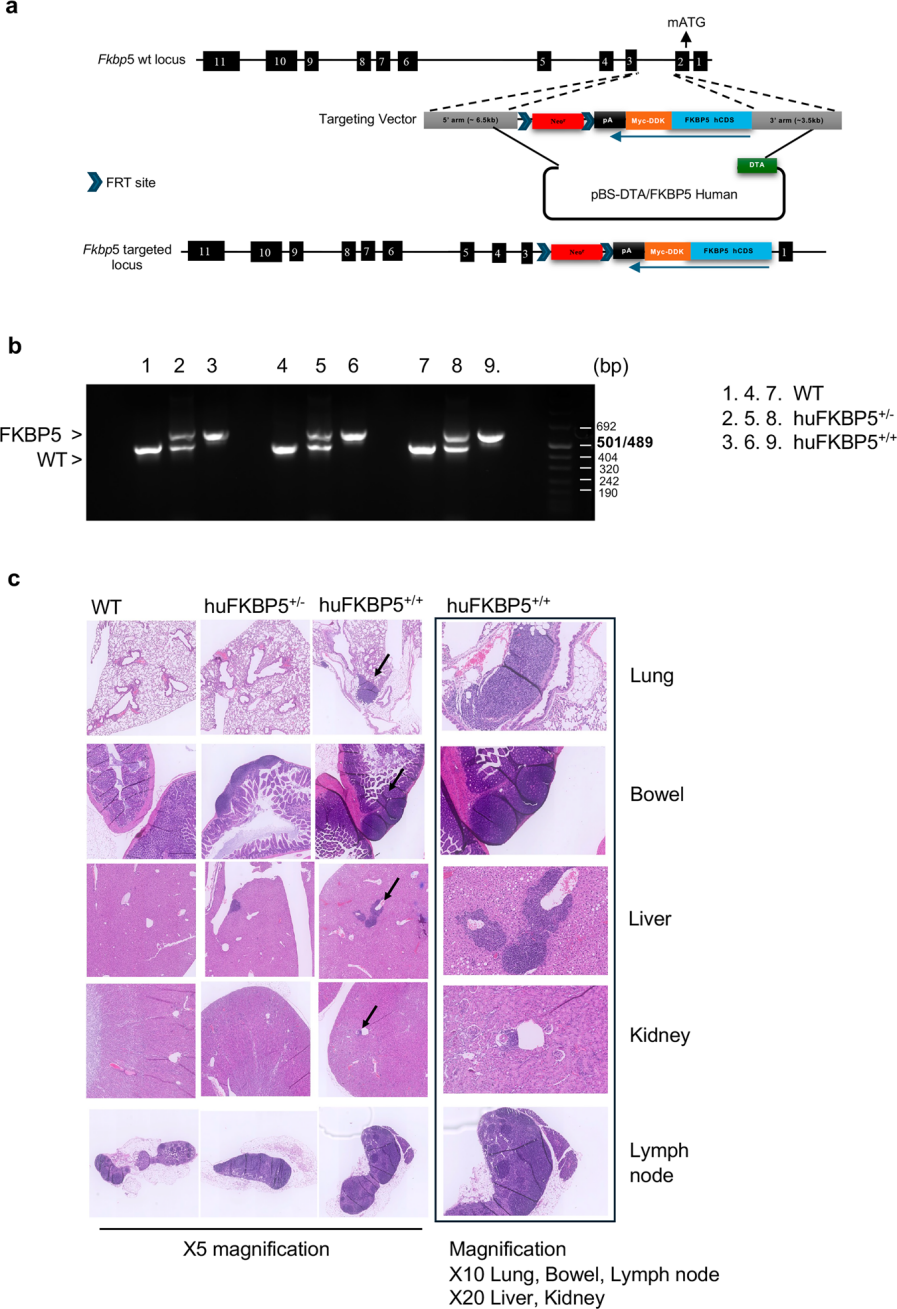
Genotype and histopathology of the humanized FKBP5 mice. **a** Targeting vectors modifying murine embryonic stem cells through homologous recombination. The targeting vector contains expression cassettes of the human *FKBP5* coding sequence (CDS), flanked by sequences that serve as homology arms necessary for homologous recombination in stem cells. Through homologous recombination, exon 2 of the murine *FKBP5* gene is replaced, starting from the ATG, with the cDNA of human isoform 1 (1371 bp), which is fused to a FLAG tag followed by a polyadenylation signal. **b** PCR genotyping of the wild type, hu*FKBP5*^+/-^ and hu*FKBP5*^+/+^ strains. Agarose gel electrophoresis revealed bands corresponding to the amplicons of three wild-type mice (lanes 1, 4, and 7), three heterozygous mice (lanes 2, 5, and 8) and three homozygous mice (lanes 3, 6, and 9). The amplicon of the murine WT *Fkbp5* gene appears as the lower band of 469 bp, and the amplicon of the human *FKBP5* gene appears as the upper band of 579 bp. Lanes 2, 5, and 8 show both amplicons. **c** Histological comparison across different genotypes (WT, hu*FKBP5*^+/-^and hu*FKBP5*^+/+^) of lung, bowel, liver, kidney, and lymph node tissues. Hematoxylin and eosin staining revealed an increasing presence of perivascular lymphoid aggregates (arrow). Distortion of the lymph node architecture is observed in hu*FKBP5*^+/+^. The amount of inflammatory infiltrate was assessed using a semi-quantitative scoring system based on the absolute number of inflammatory foci per tissue section. Scores were defined as follows: absent to mild (0–1 inflammatory foci) = WT; moderate (approximately 2–3 inflammatory foci) = hu*FKBP5*^+/-^; severe (>3 inflammatory foci) = hu*FKBP5*^+/+^.

To generate a knock-in mouse in which only the full-length isoform is expressed, we cloned the canonical isoform of human *FKBP5* into the second exon of the mouse gene in frame with the methionine start codon (Fig. 1a). In this way, all possible variants derived from alternative splicing events are abolished.

The heterozygous mice (hu*FKBP5*^+/-)^ were fertile, but the genotypes of their progeny did not adhere to the Mendelian ratio, with a very small number of homozygous mice (hu*FKBP5*^+/+^) bearing two alleles of the knock-in gene. The rare homozygous mice were not fertile. Compared with their respective wild-type (WT) counterparts, Hu*FKBP5*^+/-^ and hu*FKBP5*^+/+^ animals did not exhibit any signs of distress. Evaluations of offspring from crosses between hu*FKBP5*^+/-^ heterozygotes (based on the following parameters: weight and growth, litter monitoring, general appearance, coat condition, behavior, clinical signs, and litter size) revealed no critical issues, except for the low frequency of hu*FKBP5*^+/+^ mice and their sterility. Specifically, 3 out of 91 homozygous hu*FKBP5*^+/+^ animals were born. The three hu*FKBP5*^+/+^mice obtained (Fig. 1b; lanes 3, 6, 9) were tested for breeding both among themselves and with WT animals; despite showing no signs of distress, no offspring were produced.

Histopathological examination of hu*FKBP5*^+/+^ mice at sacrifice revealed extensive lymphocyte infiltration in various organs, lungs, intestinal walls, liver, kidney, and lymph nodes (Fig. 1c). Histological comparison of lung, bowel, liver, kidney, and lymph node tissues across different genotypes, i.e., WT, hu*FKBP5*^+/-^ and hu*FKBP5*^+/+^, revealed an increasing presence of perivascular lymphoid aggregates (arrow) along with distortion of the lymph node architecture in hu*FKBP5*^+/+^ mice (Fig. 1c). Supplementary information, Fig. S1 shows human FKBP51 expression in the PBMCs of heterozygous and homozygous mice, with expression confirmed at the transcript (Fig. S1a) and protein (Fig. S1b) levels.

**Fig. 2:**
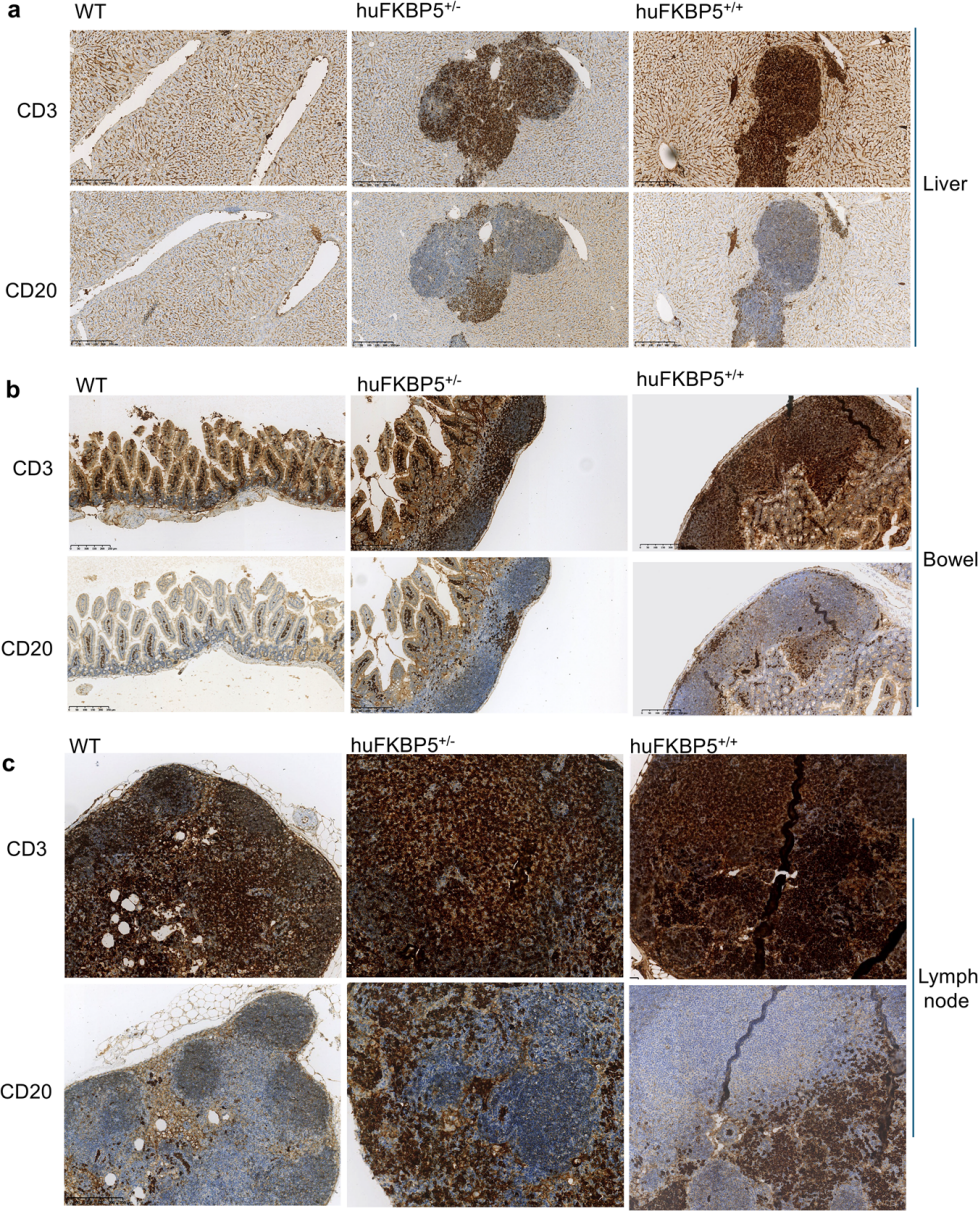
CD3 and CD20 immunohistochemical staining of human FKBP5 mouse tissues. **a** Immunohistochemical staining of liver tissue for CD3 and CD20 across genotypes (WT, hu*FKBP5*^+^/^-^, and hu*FKBP5*^+/+^) at 10× magnification. WT liver sections show no inflammatory infiltrate around the central vein. In contrast, both hu*FKBP5*^+^/^-^ and hu*FKBP5*^+/+^ genotypes display pericentrovenular inflammatory aggregates. Each image shows a single representative inflammatory aggregate from hu*FKBP5*^+^/^-^ or hu*FKBP5*^+/+^ liver sections. A marked predominance of T lymphocytes (CD3^+^) over B lymphocytes (CD20^+^) is observed. Notably, the T/B cell ratio differs between mutant genotypes: hu*FKBP5*^+^/^-^ mice exhibit approximately 80% CD3^+^ and 20% CD20^+^ cells, whereas hu*FKBP5*^+/+^ homozygotes show a more pronounced T-cell skewing, with approximately 95% CD3^+^ and 5% CD20^+^ lymphocytes. **b** Immunohistochemical staining of intestinal tissues for CD3 and CD20 across genotypes: hu*FKBP5*^+/-^ and hu*FKBP5*^+/+^ (×10 magnification). WT intestinal sections show no abnormal inflammatory infiltrate within the intestinal wall. In contrast, both hu*FKBP5*^+/-^ and hu*FKBP5*^+/+^ genotypes display inflammatory aggregates with a marked predominance of T lymphocytes (CD3^+^) over B lymphocytes (CD20^+^). The T/B cell ratio differs between mutant genotypes: hu*FKBP5*^+/-^ mice exhibit approximately 80% CD3^+^ and 20% CD20^+^ cells, whereas hu*FKBP5*^+/+^ homozygotes show approximately 90% CD3+ and 10% CD20^+^ lymphocytes, indicating a slightly less pronounced T cell skewing compared to hepatic infiltrates. **c** upper, Staining of lymph nodes for CD3 (×10 magnification) shows intense coloration in the interfollicular zones with partial colonization of follicular areas in WT mice, while in mutant mice the staining shows intense coloration in the interfollicular zones with more pronounced colonization of follicular areas (hu^FKBP5^^+/-^mice), covering almost the entire lymph node (hu*FKBP5*^+/+^). lower, Staining of lymph nodes for CD20 (×10 magnification) shows partial localization in the follicular zones in WT mice whereas, in mutant mice, CD20 staining is weaker, showing minimal (hu*FKBP5*^+/-^mice) to absent (hu*FKBP5*^+/+^) localization in follicular zones and greater presence in interfollicular areas.

CD3 and CD20 immunostaining of liver sections revealed a higher abundance of T lymphocytes (CD3⁺) than B lymphocytes (CD20⁺) within the nodular aggregates. Figure 2a shows a representative lymphoid aggregate from hu*FKBP5*^+/-^ or hu*FKBP5*^+/+^ surrounding the liver central vein. Similarly, the staining of intestinal tissues revealed an increased presence of CD3 lymphocytes compared with that of CD20 lymphocytes in aggregates within the intestinal wall of hu*FKBP5*⁺/⁻ and hu*FKBP5*⁺/⁺ mice (Fig. 2b).

CD3 and CD20 immunohistochemistry of the lymph nodes revealed distinct patterns across different genotypes (Fig. 2c). In WT mice, CD3 staining was strong in interfollicular zones (Fig. 2c, upper), whereas CD20 (Fig. 2c, lower) was localized to follicles. In hu*FKBP5*^+/-^ mice, CD3 staining remained strong in interfollicular zones and extended into follicles (Fig. 2c, upper); CD20 staining revealed reduced follicular localization and increased interfollicular localization (Fig. 2c, lower). In hu*FKBP5*^+/+^mice, CD3 was intense in both interfollicular and follicular areas (Fig. 2c, upper), whereas CD20 was largely absent from follicles and concentrated in interfollicular zones (Fig. 2c, lower).

**Fig. 3:**
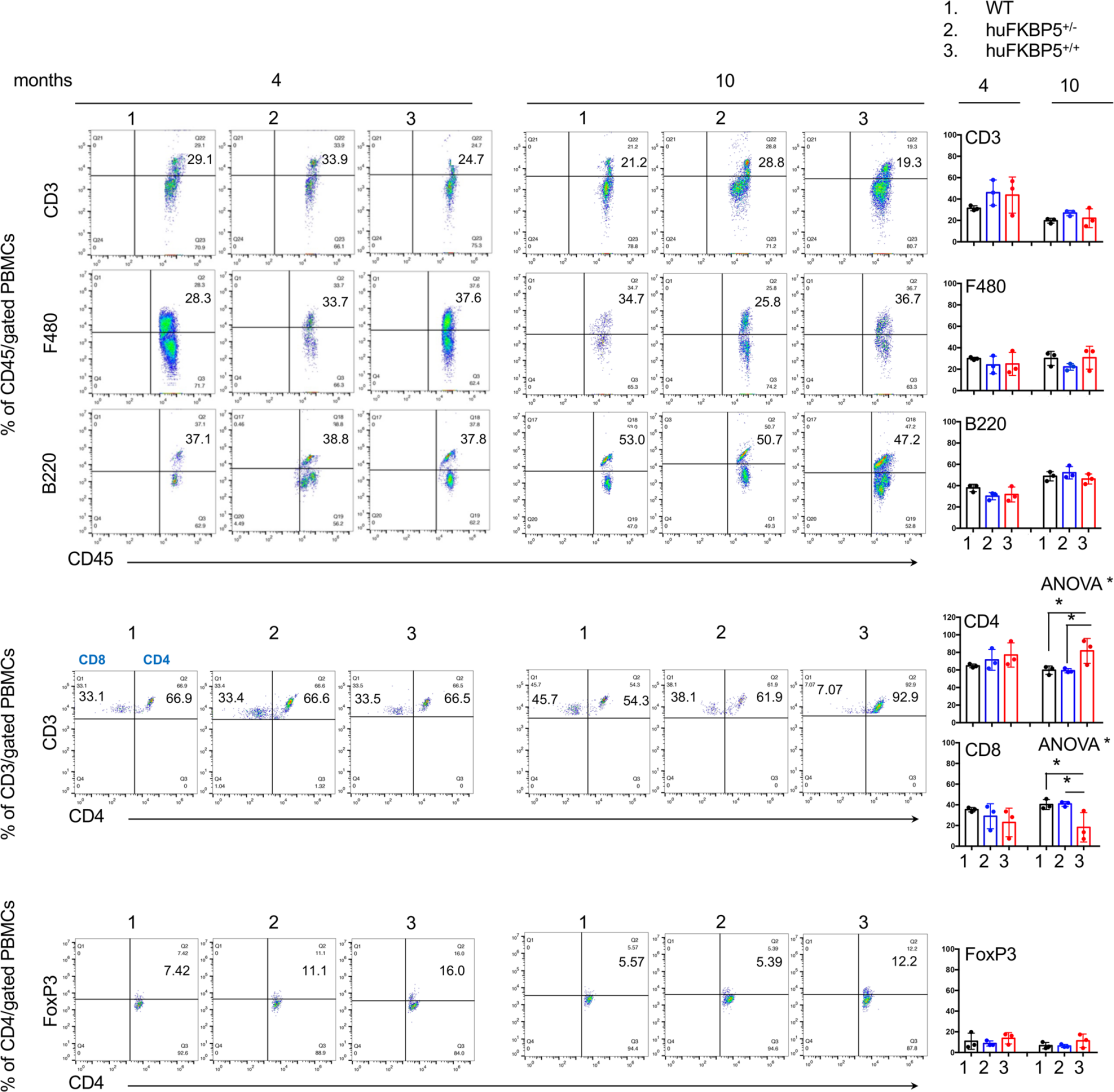
PBMC immunophenotyping of humanized FKBP5. Peripheral blood was collected by retro-orbital bleeding from humanized *FKBP5* mice at 4 and 10 months of age. PBMCs were isolated (*n* = 3 mice per group) and analyzed by multiparametric flow cytometry using the indicated clusters of differentiation (CD) markers. Representative flow cytometry dot plots are shown for all immune cell populations. Live PBMCs were first gated on CD45⁺ cells. The frequencies of CD3 T cells, F480 myeloid cells, and B220 B cells are expressed as percentages of total CD45⁺ PBMCs. CD4 and CD8 T-cell subsets are reported as percentages within the CD3/CD45 PBMC population. Tregs were identified as Foxp3⁺ cells within the CD3/CD4 gate and are expressed as percentages of CD4 T cells. Data are presented as mean ± SD.

Peripheral blood immunophenotyping was performed at 4 and 10 months of age (Fig. 3). At 10 months of age, hu*FKBP5*^+/+^ PBMCs presented a CD4^+^/CD8^+^ T-cell distribution skewed toward CD4^+^ T cells compared with that of WT and hu*FKBP5*^+/-^ PBMCs. No other significant differences in the counts of B cells, T cells, Tregs, or monocytes were detected among the three groups of mice.

### Hu*FKBP5* in mice has a remarkable ability to limit the growth of melanoma tumors

**Fig. 4:**
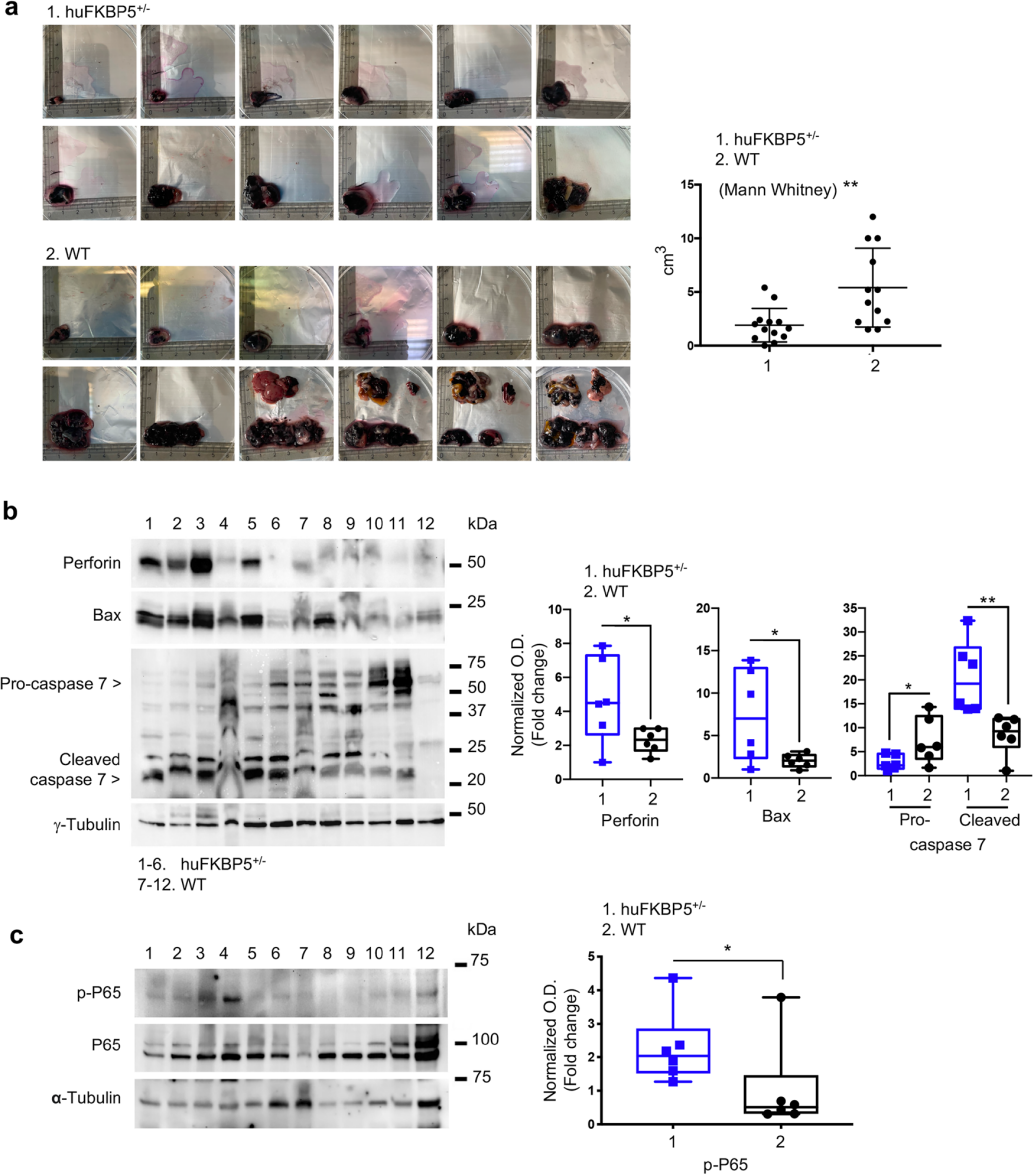
In Hu*FKBP5*-treated mice, melanoma growth is inhibited. **a** Tumors excised were photographed and measured. The graph on the right represents the volume measures. **b** Western blot analysis of perforin, Bax and active Caspase-7 expression levels in tumors from hu*FKBP5* [1–6] and WT [7–12] mice. γ-Tubulin was used as a loading control. Densitometric analysis of the bands was performed via ImageJ 1.42q for Macintosh. The full gels are shown in the Supplemental Material. **c** Western blot assay of phospho-p65 (RelA) expression in protein extracts from PBMCs isolated from hu*FKBP5* [1–6] and WT [7–12] mice at the time of tumor excision. Phospho-p65 levels were quantified and normalized to total p65. Full lenght western blot are shown in the Supplemental Material.

To investigate the immune response in a relevant context, we employed a syngeneic tumor model by subcutaneously injecting murine B16F10 melanoma cells. Owing to the markedly low birth rates of homozygous mice, only heterozygous mice, hereafter referred to as hu*FKBP5*, were used. Assessment of tumor growth revealed that tumors from hu*FKBP5* mice were significantly smaller, averaging 2.0 ± 0.4 cm³, than tumors from WT mice, which measured 5.4 ± 1 cm³ (*p* = 0.009, *N* = 12) (Fig. 4a). Consistent with the reduced tumor burden, western blot analysis of tumor lysates from hu*FKBP5* mice revealed elevated levels of perforin relative to those in WT tumors, indicating enhanced CTL activity (Fig. 4b). This heightened cytotoxic response was further supported by increased cleavage of caspase‑7 and elevated expression of the proapoptotic protein Bax (Fig. 4b), suggesting activation of a proapoptotic, immune-mediated antitumor response.

At the time of tumor excision, a western blot assay of protein extracted from peripheral blood immune cells showed elevated phospho-p65 levels in hu*FKBP5* mice compared with WT mice (Fig. 4c), indicating activation of the NF-κB pathway and reflecting a heightened immune activation state. Immunophenotypic analysis revealed no significant quantitative alterations in peripheral blood immune cell composition, between WT and hu*FKBP5* mice (Supplementary Fig. S2a). However, hu*FKBP5* mice exhibited signs of CD3⁺/CD4⁺ T-cell activation (Supplementary Fig. S2b, c), as evidenced by increased expression of the activation markers CD25 and MHC class II compared with WT controls.

### Enhanced caspase-mediated pyroptotic-like cell death in melanomas from hu*FKBP5* mice

**Fig. 5:**
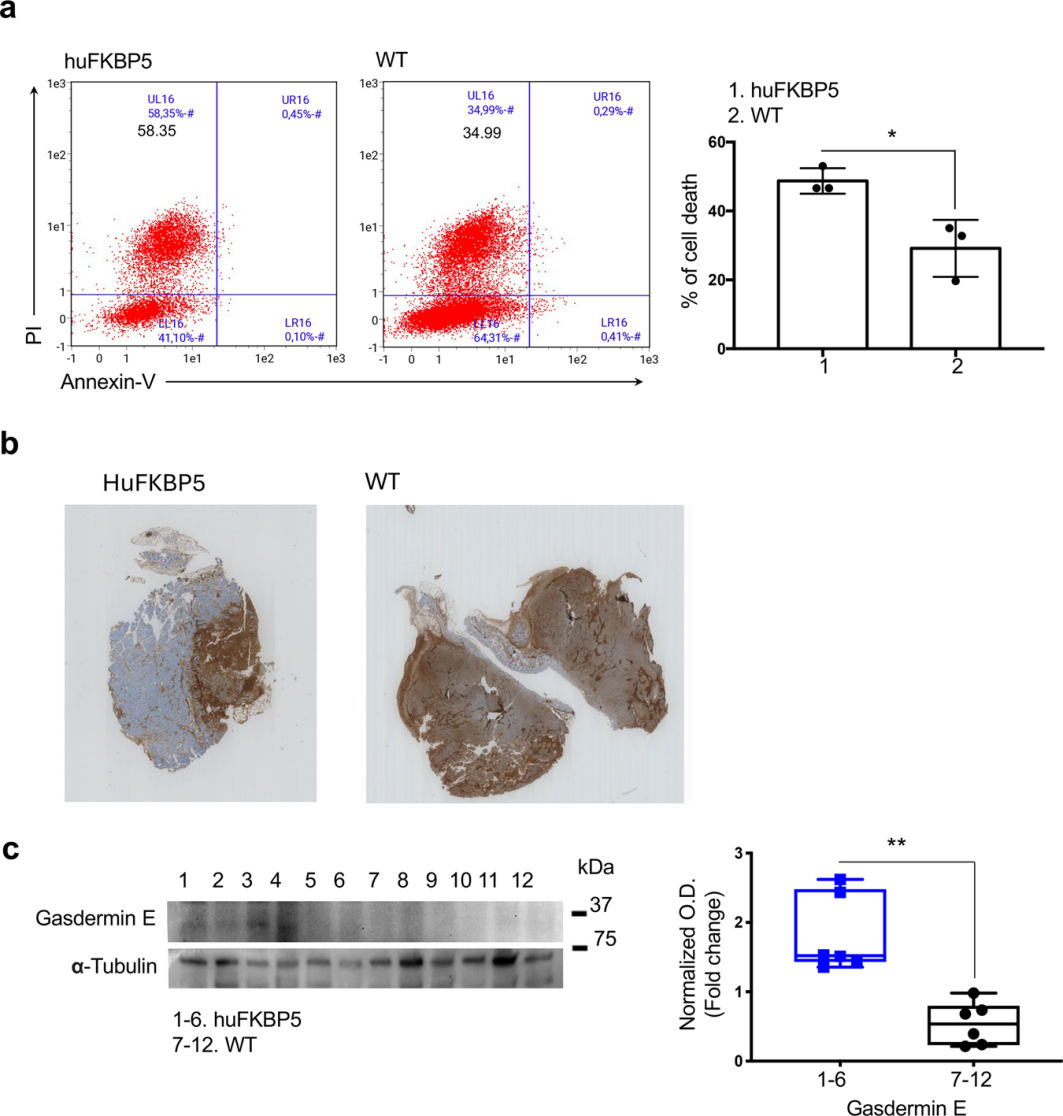
Analysis of melanoma tumor cell death and pyroptosis. **a** B16 melanoma cells were cocultured with PBMCs from WT or hu*FKBP5* mice for 24 h, and cell death was analyzed by flow cytometry. After removal of cells in suspension, adherent cells were stained with Annexin V and PI. CD45 staining was used to exclude contaminating PBMCs from the analysis. Representative flow cytometry plots are shown along with mean values ± SD (*N* = 3). **b** Immunohistochemical staining for cleaved caspase-3; panoramic histological views of melanoma tumors from hu*FKBP5* mouse or WT mouse. **c** Western blot analysis of melanoma tumors showing increased levels of cleaved GSDME in tumors from hu*FKBP5* [1–6] and WT [7–12] mice. α-Tubulin was used as a loading control. Densitometric analysis of the bands was performed via ImageJ 1.42q for Macintosh. The full gels are shown in the Supplemental Material.

To confirm that hu*FKBP5* mice have an increased capacity to induce tumor cell killing, B16 melanoma cells were cocultured (CC) with WT or hu*FKBP5* PBMCs and after 24 h cell death was analyzed in flow cytometry by annexin V and propidium iodide (PI) staining. Annexin V single-positive cells are indicative of early apoptosis, PI single-positive cells of necrotic cell death, and annexin V/PI double-positive cells of late apoptosis or early necrosis. As shown in Fig. 5a, B16 cells predominantly underwent necrotic cell death, which was more pronounced in CC with hu*FKBP5* PBMCs. Notably, cytotoxic T lymphocytes induce target cell death primarily through the perforin-mediated delivery of serine proteases known as granzymes [15, 16]. Granzyme B directly cleaves procaspase-3, and also procaspase-7, bypassing upstream death-receptor signaling [15, 16]. In parallel, gasdermins constitute a family of pore-forming proteins that execute pyroptosis, a necrotic form of programmed cell death [17]. Among them, gasdermin E (GSDME) is cleaved by caspase-3 [17]. Immunohistochemical staining for cleaved caspase-3 confirmed activation of this enzyme in melanoma tumors (Fig. 5b), with heterogeneous expression throughout the tissue. Overviews of the same tumor from hu*FKBP5* mouse as in 5b, are shown in Supplementary Fig. S3a, b. Low-magnification imaging (Fig. S3a) revealed intense cytoplasmic immunoreactivity surrounding necrotic foci. At higher magnification (Fig. S3b), a necrotic focus (black arrow) and cleaved caspase-3 cytoplasmic immunoreactivity were evident, with transitional zones displaying a gradient from weak (red arrow) to strong (green arrow) staining when approaching the necrotic interface, illustrating progressive activation of caspase-3 in peri-necrotic regions. Western blot analysis revealed increased levels of the cleaved GSDME fragment in melanoma tumors from hu*FKBP5* mice compared with WT controls, consistent with enhanced pyroptotic cell death (Fig. 5c).

### Hu*FKBP5* mice display immune remodeling of the TME

**Fig. 6:**
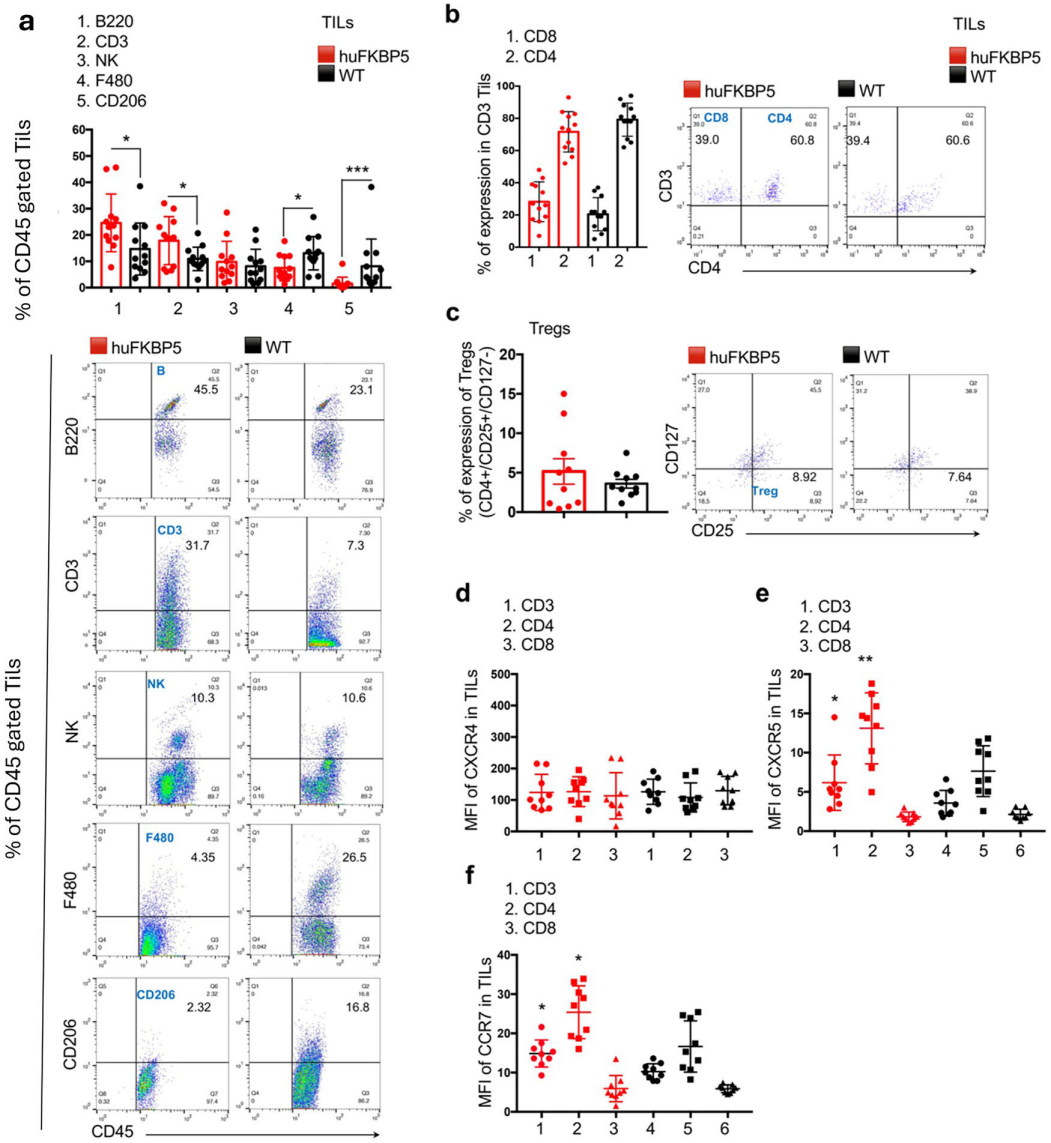
TILs analysis in WT and hu*FKBP5* mice. Tumor samples from hu*FKBP5* (red) and WT (black) mice were mechanically and enzymatically dissociated. Immune cells were enriched using CD45 magnetic beads and subsequently analyzed by multiparametric flow cytometry within the CD45⁺ leukocyte-gated population. The gating strategy used to identify all immune cell populations is shown in Supplementary Fig. S3. Flow cytometry analysis and graphical quantification of immune cell populations included: **a** T and B lymphocytes, NK cells, and macrophage subsets (F480⁺ and CD206⁺), expressed as percentages of the CD45⁺ leukocyte population (*n* = 12 mice per group); **b** CD4⁺ and CD8⁺ T cells, expressed as percentages of the CD3⁺CD45⁺ T-cell population within the tumor microenvironment; **c** Tregs, identified as CD4⁺CD127⁻ cells, expressed as percentages of the CD3⁺CD45⁺ T-cell population within the tumor microenvironment (*n* = 12 mice per group). Data are presented as mean ± SD. Chemokine receptor expression (mean fluorescence intensity, MFI) on T lymphocyte subsets of the TME: CXCR4 (**d**), CXCR5 (**e**) and CCR7 (**f**) (*n* = 9), for gating strategy and representative flow cytometry images see Supplementary Fig. S4.

We next investigated whether the heightened antitumor response of hu*FKBP5* mice was accompanied by immune remodeling of the TME. To address this point, we performed flow cytometry analysis of TILs from tumor-infiltrating lymphocytes. Our results revealed that tumors from hu*FKBP5* mice exhibited significantly greater infiltration of lymphocytes, including both B cells (Fig. 6a, 1) (24.6 ± 11% vs. 14.6 ± 9.8% in WT; % of CD45 TILs; *p* = 0.01) and T cells (17.8 ± 9.1% vs. 10.9 ± 4.4% of CD45 TILs in WT; *p* = 0.01) (Fig. 6a, 2), with reduced macrophage presence, as indicated by lower proportions of F480 (Fig. 6a, 4) (7.4 ± 4.9% vs. 13.1 ± 6.3% in WT; *p* = 0.03) and CD206 macrophages (Fig. 6a, 5) (1.5 ± 2.4% vs. 8.1 ± 10.3% in WT; *p* = 0.0005) (Fig. 6a). The relative frequencies of CD4 and CD8 subsets within the CD3 T-cell population (Fig. 6b), as well as those of Tregs (Fig. 6c) and NK cells (Fig. 6a, 3), were comparable between the two groups. The gating strategy used for the identification of CD4 and CD8 within CD3 T cells population in the TME of hu*FKBP5* or WT mice is shown in Fig. S4a, b. Supplementary Fig. S4c, d depict CD4 gating strategy and a representative analysis of Tregs. The recruitment and activation of antitumor immune cells depend largely on the expression of chemokine receptors. We measured the expression of three chemokine receptors, namely, CXCR4 (Fig. 6d), CXCR5 (Fig. 6e) and CCR7 (Fig. 6f), on TILs. CXCR4 is an atypical chemokine receptor with broad biological roles extending well beyond leukocyte trafficking. In addition to its crucial function during embryogenesis, which plays essential roles in the development of the hematopoietic, cardiovascular, and nervous systems [18], CXCR4 is involved in immune cell migration and establishing intimate contact with tumor cells [19]. However, the CXCL12–CXCR4 axis can reportedly be hijacked by tumors to create an immune-excluded microenvironment [20]. CXCR5 and CCR7 are widely associated with active and organized antitumor immune responses. They are central to lymphoid tissue organization and the formation of tertiary lymphoid structures within tumors and facilitate T-cell priming and central memory generation, both of which are essential for sustained antitumor immunity [21–23]. Our results revealed that hu*FKBP5* mouse TILs (CD3/CD4^+^cells and CD3/CD8^+^ cells) presented increased expression of CXCR5 (Fig. 6e). and CCR7 (Fig. 6f). Supplementary Fig. S5 shows the gating strategy and overlay histograms (WT vs hu*FKBP5*) of chemokine expression, which was evaluated as mean fluorescence intensity (MFI).

### Synthetic mRNA transduction of the spliced isoform into murine lymphocytes suppresses their effector function

**Fig. 7:**
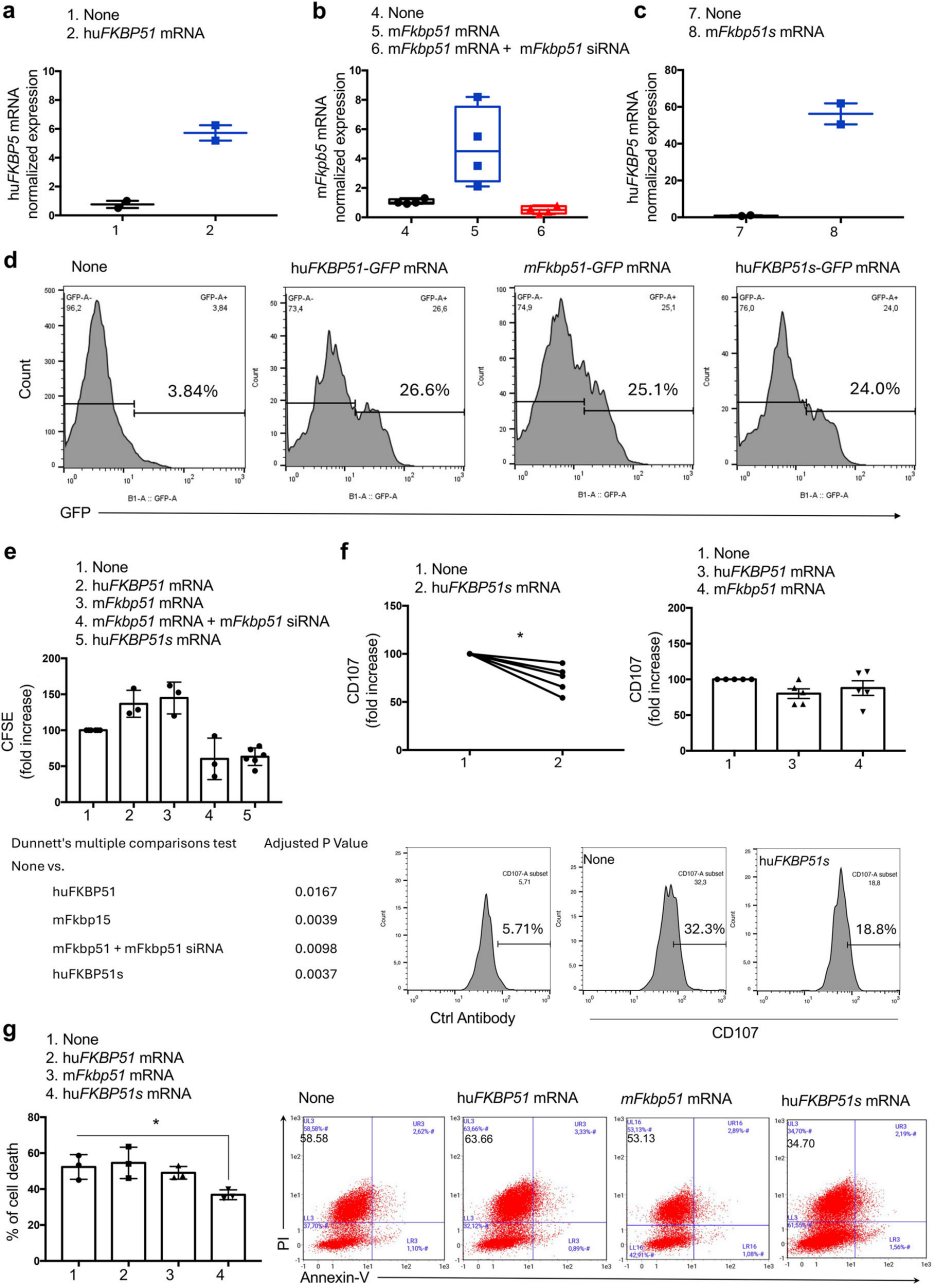
Effects of transduction of synthetic mRNAs for FKBP51 isoforms on lymphocyte activation. **a** Human (full-length) *FKBP51* mRNA levels in murine (WT) PBMCs following transfection with the corresponding RNA. **b** Murine *fkbp51* mRNA levels in untransduced or *fkbp51*-transduced PBMCs in the absence or presence of a mouse *fkbp51*-targeting siRNA. **c** Human (spliced) *FKBP51* mRNA levels in mouse PBMCs following transfection with the corresponding synthetic RNA. **d** Flow cytometry histograms of ectopic GFP expression in transduced lymphocytes. **e** Graphical representation of the proliferative response to CD3/CD28-coated beads in transduced lymphocytes, as assessed by CFSE, and compared with that of untransduced controls. **f** Measurement of CD107a surface mobilization in CD8 ^+ ^T cells cocultured with B16 melanoma cells. CD107a expression was markedly reduced in cells transfected with hu*FKBP51s* (left), whereas full-length murine or human FKBP51 had no significant effect (right). Representative flow cytometry histograms of CD107a expression in CD8⁺/CD3⁺/CD45⁺ gated-PBMCs cocultured with B16 melanoma cells, either untransduced [1] or transduced [2] with spliced human FKBP51s. **g** Measurement of cell death in B16 melanoma cells CC with WT or hu*FKBP5* PBMCs. After removal of cells in suspension, adherent cells were stained with Annexin V and PI. CD45 staining was used to exclude contaminating PBMCs from the analysis. The left panel shows mean values ± SD (*N* = 3), while the right panel displays representative flow cytometry plots of Annexin V and PI staining.

We evaluated the impact of the ectopic expression of human and murine full-length FKBP51, as well as the human spliced isoform FKBP51s, on PBMC activation. To this end, we synthesized capped mRNAs encoding each protein assembled in lipid nanoparticles, which were subsequently transduced into murine lymphocytes stimulated with CD3/CD28, and we measured their proliferation and cytotoxic effector function (Fig. 7). Figure 7a shows human FKBP51 mRNA levels in murine PBMCs following transduction with the corresponding RNA. Figure 7b shows the murine *fkbp51* mRNA levels in untransduced or *fkbp51*-transduced PBMCs in the absence or presence of a mouse *fkbp51*-targeting siRNA. Figure 7c shows the human *FKBP51* mRNA levels in mouse PBMCs following transduction with the corresponding synthetic RNA. Synthetic mRNAs were co-formulated with GFP, enabling visualization and confirmation of successful protein expression (Fig. 7d). The proliferative response of transduced lymphocytes was quantified and compared with that of untransduced controls (Fig. 7e). The overexpression of full-length human *FKBP51* significantly enhanced lymphocyte proliferation. Similarly, ectopic expression of murine full-length fkbp51 elicited a comparable increase in proliferation, which was abrogated upon targeted silencing of murine *fkbp51*, validating its functional contribution. In contrast, the expression of human isoform FKBP51s markedly suppressed lymphocyte proliferation. Collectively, these findings demonstrate the functional equivalence of the full-length FKBP51 protein across species and support a model in which FKBP51s acts as dominant-negative regulators of lymphocyte activation in murine cells.

Furthermore, we evaluated whether the expression of the two FKBP51 isoforms modulated the cytotoxic function of lymphocytes by measuring CD107a surface mobilization in CD8^+ ^T cells cocultured with B16 melanoma cells. Strikingly, CD107a expression was markedly reduced in cells transfected with hu*FKBP51s,* whereas full-length murine or human FKBP51 had no effect on CD107 modulation (Fig. 7f), highlighting the major role of the short isoform in suppressing CD8 T-cell degranulation and effector activity. Consistently, analysis of tumor cell death in B16 cocultures revealed that transduction of hu*FKBP51s* into PBMCs significantly reduced their cytotoxic killing capacity (Fig. 7g). In contrast, no significant changes in melanoma cell killing were observed in cocultures with PBMCs transduced with either human or murine FKBP51 (Fig. 7g).

## Discussion

The functional balance between the full-length canonical FKBP51 and its short variant FKBP51s may serve as a key mechanism for fine-tuning T-cell responses, particularly during the phases of expansion and homeostatic return [6]. In this study, we generated a humanized mouse engineered to express human full-length FKBP51 while preventing the production of alternative splice variants to investigate the immunological consequences of selectively losing FKBP51s while preserving FKBP51 expression.

Our findings revealed that the loss of FKBP51s has a significant effect on mouse physiology. Homozygous hu*FKBP5*^+/+^ mice are infertile and are born at a much lower frequency than expected from Mendelian inheritance. Although they exhibit no overt signs of distress, histopathological analysis revealed widespread inflammatory infiltrates with lymphoid aggregates in various organs, along with architectural disruption of the lymph nodes, indicating altered immune regulation.

PBMCs from hu*FKBP5*^+/+^ mice show a balance between CD4^+^ and CD8^+^ T cells in favor of CD4^+^ T cells. Histological examination of peripheral tissues revealed a predominance of CD3^+ ^T-cell infiltration. In particular, in the lymph nodes, strong CD3 positivity extended into both interfollicular and follicular areas, suggesting disrupted lymphoid organization.

The most striking result concerns the antitumor immune response. Heterozygous hu*FKBP5* mice exhibit important tumor control in a syngeneic melanoma model. Despite the absence of quantitative alterations in peripheral blood immune cell composition, at the time of tumor excision, hu*FKBP5* mice displayed a clear immune activation profile. In particular, T cells exhibited increased expression of activation markers, accompanied by activation of the NF-κB signaling pathway, indicating a heightened immune activation state.

Major immunological differences emerged at the level of the tumor microenvironment. Immunophenotypic analysis of the TME revealed increased lymphocyte infiltration and reduced macrophage presence, particularly a notable decrease in CD206^+^ macrophages, in contrast to those in tumors from WT mice. These findings suggest that the protective phenotype in hu*FKBP5* tumors is driven by increased recruitment of effector lymphocytes and a concomitant reduction in the number of immunosuppressive macrophages, leading to a more immunostimulatory microenvironment that can restrain tumor growth.

A strengthened antitumor microenvironment in hu*FKBP5* mice is further supported by increased expression of perforin and Bax, along with elevated levels of activated caspase-7. Immunohistochemical staining for cleaved caspase-3 revealed parallel activation in melanoma tumors. Moreover, increased GSDME cleavage in tumor lysates from hu*FKBP5* mice compared with WT controls is consistent with a pyroptotic-like mode of tumor cell death that is more pronounced in hu*FKBP5*.

Increased expression of CXCR5 and CCR7 on tumor-infiltrating lymphocytes suggests the establishment of an organized, proinflammatory tumor microenvironment conducive to immune surveillance and tertiary lymphoid structure formation [21–23].

Transduction experiments with synthetic mRNAs demonstrated functional equivalence between murine and human full-length FKBP51 in supporting lymphocyte proliferation, which is consistent with the established role of canonical FKBP51 in promoting NF-κB signaling [4]. This transcription factor is one of the central hubs controlling lymphocyte activation, which shapes the quality, magnitude, and durability of adaptive immune responses.

In contrast, FKBP51s transduction impaired murine T-lymphocyte activation and effector function, consistent with its dominant-negative activity toward full-length FKBP51 and the IKK complex [4]. In addition, based on its previously described role as a cochaperone regulating PD-L1 post-translational processing and surface expression [9, 10], FKBP51s may further restrain T-cell effector responses by reinforcing PD-1/PD-L1–mediated inhibitory signaling. Accordingly, the enhanced antitumor activity observed in hu*FKBP5* mice may result, at least in part, from disruption of this immune inhibitory axis. Although this mechanism was not directly addressed in the present study, it represents a non-mutually exclusive layer of immune regulation that warrants targeted investigation.

Collectively our findings highlight that the relative balance between FKBP51 isoforms is a critical determinant of lymphocyte functional competence. In hu*FKBP5* mice, an important regulatory constraint on canonical FKBP51 activity is relieved, which explains increased immune effector function ultimately favoring antitumor immune responses.

In conclusion, our results identify *FKBP5* alternative splicing as a central regulatory layer controlling lymphocyte activation and immune tolerance. Alterations in isoform balance have profound consequences for tumor immune surveillance, providing a rationale for therapeutic strategies aimed at modulating *FKBP5* splicing or selectively targeting FKBP51s to enhance cancer immunotherapy.

### Limitations

While our study provides novel insights into the role of FKBP51 isoforms in immune regulation and tumor control, several limitations should be acknowledged.

Although in vitro transduction experiments with synthetic RNA provide evidence implicating FKBP51s loss as a key determinant of the observed phenotypes, it was impossible to test the mouse fkbp51s isoform because of the lack of enough information about the alternative splicing events occurring in the mouse. However, another humanized *FKBP5* mouse model [7, 8], carrying the entire gene rather than only the coding sequence, does not present the developmental and fertility issues observed in our model, suggesting a strict functional similarity between human and mouse splice variants of the *FKBP5* gene.

The molecular mechanisms underlying the functions of the two FKBP51 isoforms are still not fully understood. Despite this limitation, the consistency of our findings with prior data and the immunological relevance of the observed changes lay a strong foundation for future studies.

## Materials and methods

### Animal studies, housing and treatment

The generation of a knock-in mouse model expressing only the canonical human FKBP51 isoform was commissioned to Biogem (Ariano Irpino, AV, Italy). In particular, the full-length human coding sequence was isolated from the true-ORF-Myc-DDK-tagged human *FKBP51*-transcript variant 1 purchased from OriGene Technologies (MD, USA) and inserted in frame into exon 2 of the murine *Fkbp5* gene, replacing the start codon to ensure endogenous expression while eliminating alternative splice variants. The targeting vector was constructed by recombineering technology, verified by sequencing and electroporated into embryonic stem cells (R1 cell line; strain 129X1 × 129S1). Correctly targeted clones were identified through PCR, as described in *the “Genotype PCR and qPCR”* section. For Southern blotting, genomic DNA was digested with the EcoRV enzyme. For hybridization, a probe on the 3’ arm was used. Positive ES clones were injected into morula to obtain chimeras and subsequently germline chimeras. Germline transmission was confirmed by breeding, and genotypes were validated via allele-specific PCR. Heterozygous (huFKBP5^+/-^) mice were intercrossed to generate homozygous and heterozygous cohorts. Genotyping was performed via PCR as described in *the “Genotype PCR and qPCR”* section; genotype frequencies and phenotypes, including body weight, fertility, behavior, and coat condition, were assessed as described in the *Results* section. C57BL/6 mice were purchased from Envigo (Telangana, India) and, along with mice from Biogem, were housed at the Federico II University DMMBM animal facility under controlled temperature (22 ± 2 °C), humidity (55 ± 5%), light-controlled conditions (12 h light:12 h dark cycle), ad libitum access to food and water, and pathogen-free conditions. All animal care procedures and experiments were approved by the Institutional Animal Care and Use Committee (Authorization n° 253/2020- PR and n° 966/2020-PR). All efforts were made to minimize animal suffering during the experiment. WT, hu*FKBP5*^+/-^ and hu*FKBP5*^+/+^ mice (3/group) were subjected to histopathological examination as described in the *Histopathology and immunohistochemistry* section. Retroorbital plexus blood collection was used to analyze peripheral blood cells in 4- or 10-month-old mice, which were then processed for immunofluorescence (IF), as described in the *Flow cytometry* section. For syngeneic tumor engraftment, 5 × 10^5^ B16 cells were injected subcutaneously into the right hind flanks of 12 WT and 12 hu*FKBP5*^+/-^ mice in 100 μL of 1 × PBS. Tumors were monitored every few days to avoid exceeding the maximal tumor size/burden allowed by the ethics committee. Before sacrifice, the mice were anesthetized with tiletamine-zolazepam (30 mg/kg) via intraperitoneal injection to minimize stress and discomfort. At the scheduled sacrifice time (14 days after injection), the mice were sacrificed via CO_2_ asphyxiation, and peripheral blood samples were collected from the maxillary venous sinus via a sterile lancet. Three hundred to 500 μL of peripheral blood per animal was collected in EDTA tubes, with 50 μL used for each IF, as described in the *Flow cytometry* section. Tumors were excised, stored in MACS® Tissue Storage Solution (Miltenyi, Bergisch Gladbach, Germany) to preserve the tissue before being mechanically dissociated, and then processed via a Tumor Dissociation Kit (Miltenyi #130-096-730) and gentleMACS Dissociator according to the manufacturer’s protocol. Dissociated cells, containing both tumor and immune infiltrates, were subjected to positive magnetic selection of CD45^+^ cells (CD45 Microbeads, Miltenyi) to isolate murine tumor infiltrating leukocytes (TILs), which were then processed for IF, as described in the *Flow cytometry* section, or cultured for further experiments and tumor cells, which were then processed for immunoblotting, as described in the *immunoblotting and antibodies* section.

### Histopathology and immunohistochemistry

WT, hu*FKBP5*^+/-^ and hu*FKBP5*^+/+^ mice (2 for each group) were subjected to histopathological examination. Mouse organs (lung, bowel, liver, kidney, and lymph nodes) were fixed in formalin for 24–48 h and then embedded in paraffin. Sections of approximately 4 µm were cut and stained with hematoxylin and eosin (H&E). Stained sections were observed under a light microscope, and a quantitative evaluation of inflammatory infiltrates and their localization within various organs was performed. Moreover, immunostaining for lymphocyte markers (CD3, CD4, CD8, and CD20) was performed as follows. Formalin-fixed, paraffin-embedded sections were cut and placed on positively charged glass slides. The slides were then deparaffinized and rehydrated with reaction buffer. Heat-induced epitope retrieval (HIER) was performed via PT-LINK (Agilent, Santa Clara, CA, United States) for 60 min at 100 °C. Immunohistochemistry was carried out on an Agilent ASLINK48 automated slide stainer. The samples were incubated with primary mouse monoclonal antibodies against CD20, CD3, CD4, and CD8 according to the manufacturer’s instructions. Visualization was achieved via the EnVision FLEX+ Mouse, High pH (Link) (Code K8002) detection system (Agilent). The samples were then coverslipped and visualized under a light microscope. For IHC of cleaved caspase-3, the rabbit polyclonal antibody against cleaved caspase-3 (Asp175, #9661 Cell Signaling, Danvers, Massachusetts USA) 1:100 was used to stain tumor sections of 4-mm thickness from formalin-fixed, paraffin-embedded blocks.

### Genotype PCR and qPCR

Correctly targeted ES clones were identified through PCR at the 5’arm, and Southern blotting at the 3’arm was performed on the genomic DNA via the following oligonucleotides:

Fw: 5′-GCTGCGGCTGTTCTTAGACCTG-3′ external to the homology arm;

Rev: 5′-CCATCTTGTTCAATGGCCGATCCC-3′ internal to the expression cassette.

Genotyping was performed via PCR by using oligonucleotides for specific recognition by WT, hu*FKBP5*^+/-^ and hu*FKBP5*^+/+^ mice. The gene-specific primers used were as follows:

Fw (WT and Hu): 5′-ATGGGTGTGGCTGGTTATGG-3′ (murine intron 1)

Rev (WT): 5′-TCCATTGTTACTGGTGCCCTC-3′ (murine exon 2)

Rev (Hu): 5′-ATCGGCGTTTCCTCACCATT-3′ (human CDS)

All oligos were designed and purchased from Eurofins (Luxembourg, Luxembourg), and the PCR cycling conditions consisted of 95 °C for 1 min and 35× (95 °C for 15 s, 60 °C for 15 s, and 72 °C for 30 s). PCR amplification was carried out via Wonder Taq DNA Polymerase (Euroclone, Pero, Milan, Italy) according to the manufacturer’s protocol. The PCR products were analyzed via agarose gel electrophoresis to determine the genotype along with a molecular ladder of DNA8 ranging from 19 to 1114 bp (Merck, Darmstadt, Germany).

Total RNA was extracted from cells with TRIzol (Thermo Fisher Scientific, Waltham, MA, United States). Each RNA sample was used for cDNA synthesis via iScript Reverse Transcription (Bio-Rad, Hercules, CA, United States). Relative gene expression was quantified via qPCR via the 2^-DDCt^ comparative method with SsoAdvancedTM SYBR Green Supermix (Bio-Rad) and specific qPCR primers. The oligo primers used for hu*FKBP5* were purchased from Qiagen (validated QuantiTect primers, San Diego, CA, USA). Other oligo sequences are reported:

m-*Fkbp5*_Fw: 5′-AAGAGAGAACTGTGTGCCGA-3′,

m-*Fkbp5*_Rev: 5′-CCCTCTCCTGCCTCTCTAGT-3′,

m-*Gapdh*_Fw: 5′-GTATGACTCCACTCACGGCAAA-3′,

m-*Gapdh*_Rev: 5′-TTCCCATTCTCGGCCTTG-3′,

m-*β-Act*_Fw: 5′-GATCAAGATCATTGCTCCTCCTGA-3’

m-β-*Act*_Rev: 5′-AGGGTGTAAAACGCAGCTCA-3’

### Cell culture, transfection and reagents

The murine melanoma B16F10 cell line was purchased from ATCC (ATCC-CRL-6475; Manassas, Virginia, USA). B16F10 cells were cultured in DMEM (Corning, New York, United States) supplemented with 10% heat-inactivated FBS (Corning), 200 mM glutamine (Corning), and 100 U/ml penicillin‒streptomycin (Corning). The B16F10 cell line was tested for mycoplasma after every thaw using a PCR-based method suitable for the detection of 11 Mollicutes and capable of detecting all Mycoplasma species, as indicated by Molla Kazemiha and colleagues [24]. After thawing, the cells were used in a range of passage numbers from the 4th to the 10th–12th to maintain the safe identity of the cell line. Murine peripheral blood mononuclear cells (PBMCs) were separated via differential centrifugation via a Ficoll‒Hypaque density gradient (Histopaque-1077, Merck). PBMCs and isolated tumor-infiltrated leukocytes (TILs) were maintained in RPMI-1640 medium (Corning) supplemented with 10% heat-inactivated fetal bovine serum (FBS; Corning), 200 mM glutamine (Corning), and 100 U/ml penicillin‒streptomycin (Corning). PBMC or TIL activation was conducted by stimulating cells with Dynabeads™ Mouse T-Activator CD3/CD28 (4.5 μm, Thermo Fisher Scientific) for 16 h. For coculture experiments, B16F10 tumor cells were seeded in 12-well plates (100.000 cells/well). After 24 h, activated murine PBMCs were added at a tumor-to-lymphocyte ratio of 1:10. Following an additional 24 h of coculture, lymphocytes were collected together with the medium by gentle pipetting and subsequently processed for IF, and B16 were subjected to cell death analysis as detailed in the *flow cytometry* section.

For silencing, specific siRNAs (s66095, Thermo Fisher Scientific) and Hs_*FKBP5*_5 FlexiTube siRNAs (SI02780372, Qiagen) were used for murine Fkbp51 and human FKBP51 silencing, respectively, and were transfected with metafectene (Biontex, Munich, Germany) according to the manufacturer’s instructions. To achieve FKBP51 isoform overexpression, FKBP in all its isoforms and variants as well as eGFP mRNAs were synthetically generated via in vitro transcription as previously reported [25]. DNA templates were generated by cloning synthetic dsDNA into a plasmid downstream of the T7-AGG promoter and upstream of the 3’-UTR, which consists of Tle5 and mtRNR UTRs. A segmented 100 nt polyA tail was cotranscriptionally incorporated upstream of a type II restriction site into the DNA plasmid template. In accordance with the T7 Flashscribe manufacturer’s instructions (CellScript, Madison, WI, United States), upon plasmid linearization, 1 µg of the template was transcribed. mRNA IVT was obtained by replacing 100% of the uridine with N1-methylpseudouridine-5’-triphosphate and fully capping with CleanCap AG (3’ OMe) (Trilink, San Diego, CA, United States). The in vitro transcription (IVT) mixture was incubated for 2 h at 37 °C. DNase was then added, and the mixture was incubated for 15 min. IVT RNAs were then purified via affinity chromatography via oligo dT resin according to the manufacturer’s instructions (Thermo Fisher). The purity of the RNA and the monodispersed peak were confirmed via capillary electrophoresis via TapeStation and then quantified via Qbit. The purified mRNAs were then formulated in lipid nanoparticles (LNPs) with the same composition as the SpikeVax vaccine (SM-102, DSPC, cholesterol, and PEG lipids at a 50:10:38.5:1.5 ratio). This formulation process was performed via the Ignite NanoAssemblr microfluidic apparatus by Precision Nanosystem-Cytiva.

### Flow cytometry

Murine whole blood and TILs were stained for analysis via flow cytometry as follows. Five to ten microliters of antibody recognizing typical cluster differentiation (CD) was added to 50 μl of whole blood or 1 × 10^6^ TILs and incubated for 30 min in the dark at 4 °C. For whole blood samples, red blood cell lysis was performed after antibody incubation by adding 450 μL of 1X FACS Lysing Solution (BD Pharmingen, San Jose, CA, United States), followed by gentle vortexing and incubation for 15 min in the dark at room temperature. All the samples were washed with flow cytometry staining buffer before further processing. For intracellular staining, 200 μL of fixation/permeabilization buffer (BD-Pharmingen Cytofix/Cytoperm Kit) was added to each tube. The samples were incubated for 20 min in the dark at 4 °C. For IF, the following antibodies were used: anti-mouse-F4/80-PE-Vio770 (Clone REA126, Miltenyi Biotec), anti-mouse-CD45-VioGreen (Clone REA737, Miltenyi Biotec), anti-mouse-CD45R/B220 FITC (Clone RA3-6B2, Biolegend, San Diego, CA, United States), anti-mouse-CD206 FITC (Clone MR5D3, Thermo Fisher Scientific), anti-mouse-CD3-VioBright B515 (Clone REA641, Miltenyi Biotec), anti-mouse-CD3-PerCP-Vio® 700 (Clone REA641, Miltenyi Biotec), anti-mouse-CD4 PE-Vio 770 (clone REA604, Miltenyi Biotec), anti-mouse-CD4 PE (clone GK1.5, Invitrogen eBioscience), anti-mouse-CD8a-PE-Vio770 (Clone REA601, Miltenyi Biotec), anti-mouse-CD14-APC-Vio770 (Clone REA934, Miltenyi Biotec), and anti-mouse-NK1.1-APC (Clone REA1162, Mil Isotype-conjugated control antibodies were used to assess nonspecific binding). PBMC proliferation in coculture experiments (see the *Cell Culture, Transfection and Reagents* section) was assessed via the CFSE assay (Thermo Fisher Scientific) following the manufacturer’s instructions. Briefly, the cells were washed twice with PBS, resuspended at 5×10⁶ cells/mL in prewarmed PBS, and labeled with 1 μM CFSE for 10 min at room temperature in the dark. Labeling was quenched with cold complete medium (10% FBS) and incubated on ice for 5 min. The cells were then washed three times with complete medium and analyzed via flow cytometry. Cell death was measured using propidium iodide (PI) in double staining with annexin V-FITC. Briefly, B16 melanoma cells were harvested 24 h after coculture (CC) with WT or hu*FKBP5* PBMCs, resuspended in 100 μl of binding buffer (10 μM Hepes/NaOH pH 7.5, 140 μM NaCl, and 2.5 μM CaCl2) containing 1 μl of annexin-V-FITC (Pharmingen/Becton Dickinson, San Diego, CA, USA) and 10 μl of PI (Pharmingen/Becton Dickinson), and incubated for 15 min at room temperature in the dark. Flow cytometry acquisition was performed using a MACSQuant Analyzer 10 (Miltenyi Biotec, Gladbach, NWR, Germany) and data were analyzed using FlowJo software (Pharmingen/Becton Dickinson). Flow cytometry acquisition was performed via a MACSQuant Analyzer 10 flow cytometer (Miltenyi Biotec) or a BD Facs Celesta (BD Pharmingen). All the data were analyzed via FlowJo software.

### Immunoblotting and antibodies

To obtain whole lysates, the cells were collected, and the cellular pellets were homogenized in modified RIPA buffer (50 mM Tris-HCl pH 7.5, 125 mM NaCl, 1% NP-40, 0.25% Na-deoxycholate, 1 mM Na-fluoride, 1 mM Na-orthovanadate, 1 mM phenylmethanesulfonylfluoride (PMSF), 1 mM dithiothreitol (DTT), and protease inhibitor cocktail). After 30 min of incubation on ice, the lysates were centrifuged at 14,000 rpm for 15 min to remove cell debris, and the supernatants were saved for immunoblotting. The protein concentration was determined via the Bradford protein assay (Bio-Rad), which measures the absorbance at 595 nm. The total protein content of the cell lysates was equalized, and the final volume was adjusted with water and Laemmli sample buffer (LB). The samples were denatured for 5 min at 95 °C, loaded on a 10% T SDS‒PAGE gel and transferred onto a methanol-activated PVDF membrane (Immobilon-P, Millipore, Merck). The membranes were incubated with the corresponding primary antibody at 4 °C overnight. Primary antibodies against the following proteins were diluted as follows: Perforin (Clone δG9, mouse monoclonal, Invitrogen-Thermo Fisher Scientific) 1:500; Bax (B9, sc-7480, mouse monoclonal, Santa Cruz Biotechnology, Dallas, TX, United States) (1:500); Caspase-7 (C7724, rabbit polyclonal, Sigma-Aldrich-Merck) 1:3000; DFNA5/GSDME (EPR19859, rabbit monoclonal, ab215191 Abcam) 1:300; α-Tubulin (5M5, Monoclonal Antibody E-AB-20036, Elabscience, Houston, TX, United States) 1:5000; γ-Tubulin (GTU-88, mouse monoclonal, Sigma-Aldrich/Merck) 1:5000; GAPDH (D4C6R, Mouse Monoclonal Antibody #97166 1:5000; FKBP51 (NB100-68240, rabbit polyclonal, Novus Biological, Abingdon, UK) (1:3000); phospho-NF-κB p65 (Ser536) (93H1, #3033, rabbit monoclonal, Cell Signaling) 1:500, NFκB p65 (F-6, sc-109, rabbit polyclonal, Santa Cruz Biotechnology) 1:1000; and Vinculin (G-11, sc-55465, mouse monoclonal, Santa Cruz Biotechnology) (1:3000).

### Statistical analysis

The results reported are the mean and standard deviation obtained from independent experiments. Statistical analysis was performed by Student’s *t* test and one-way ANOVA, using Prism GraphPad 7.0a. A *p* ≤ 0.05 was considered statistically significant. *P < 0.05, **P < 0.01, ***P < 0.001, ****P < 0.0001.

## Supplementary information

Supplementary

## Data Availability

All data generated or analyzed during this study are included in this published article and its supplementary information files.
