# Supplementary material for: FKBP5 isoforms shape immune pathways related to tumor tolerance
Source: Cell Death Discov. 2026 Mar 26;12:233. doi: 10.1038/s41420-026-03047-5 (PMC13184302; doi:10.1038/s41420-026-03047-5)

Supplementary information

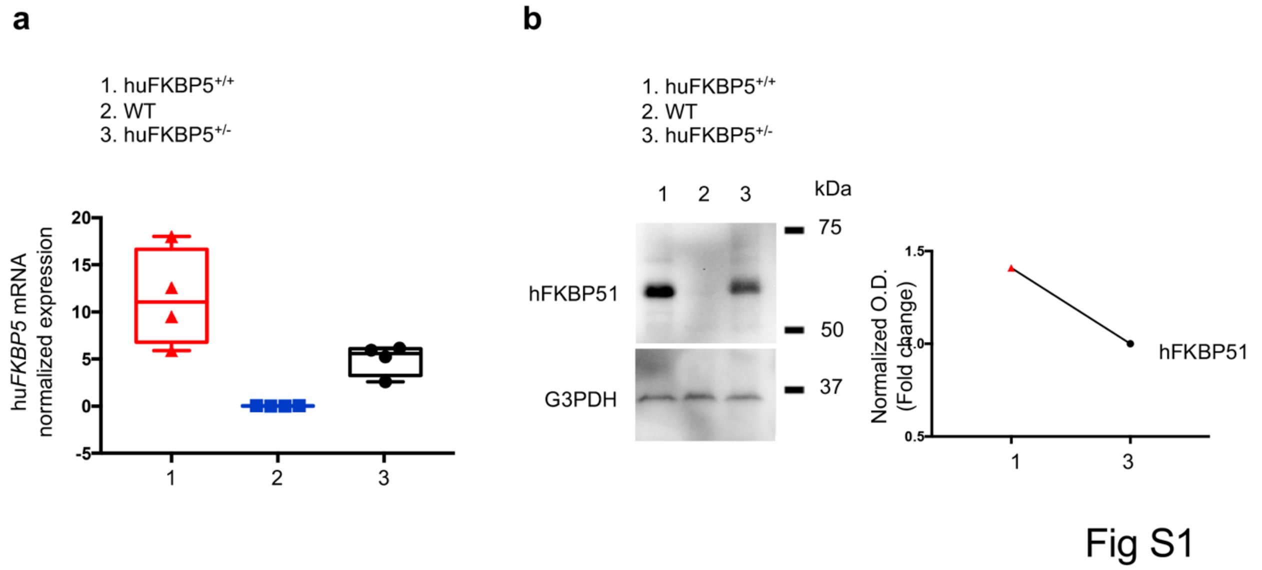

Figure S1. Human FKBP51 expression in PBMCs of humanized mice. a, Relative normalized expression levels of human FKBP51 transcript, in total RNA extracted from PBMCs of WT, huFKBP5+/- and huFKBP5+/+ mice. **b**, Western blot assay of human FKBP51 (hFKBP51) expression level in lysates obtained from PBMCs of WT, huFKBP5+/- and huFKBP5+/+ mice. Full lenght western blots follow:

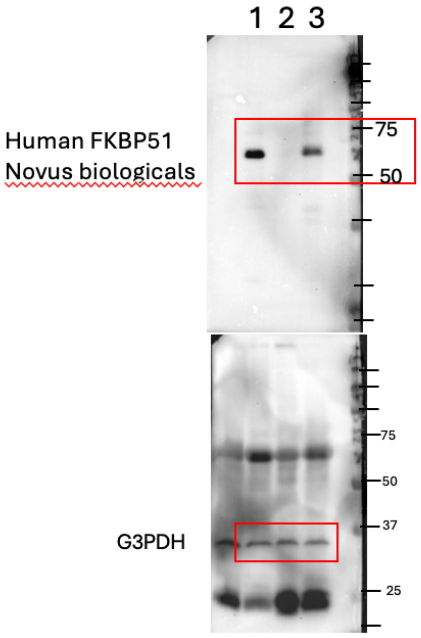

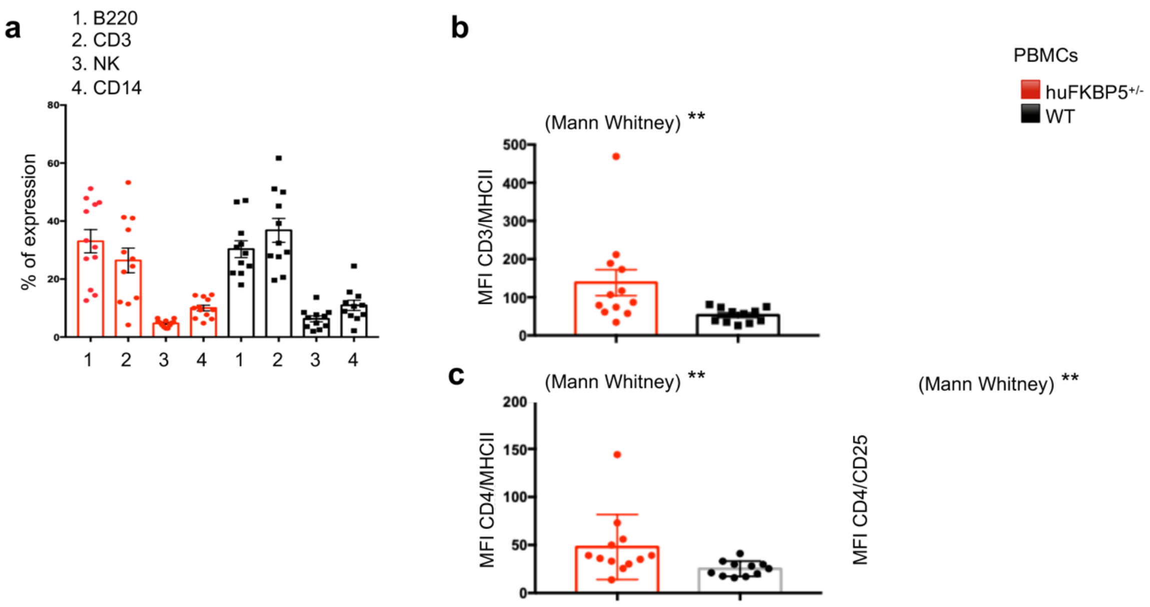

Fig S2

Figure S2. Immunophenotypic analysis of PBMCs. No significant differences in B, T, NK, or monocyte counts were observed between WT (black) and huFKBP5 (red) mice (a). huFKBP5 mice show CD3 lymphocyte activation (b), with increased MHC class II expression compared to WT controls. Similarly, CD3/CD4 lymphocytes in huFKBP5 mice display elevated MHC class II and CD25 expression (c).

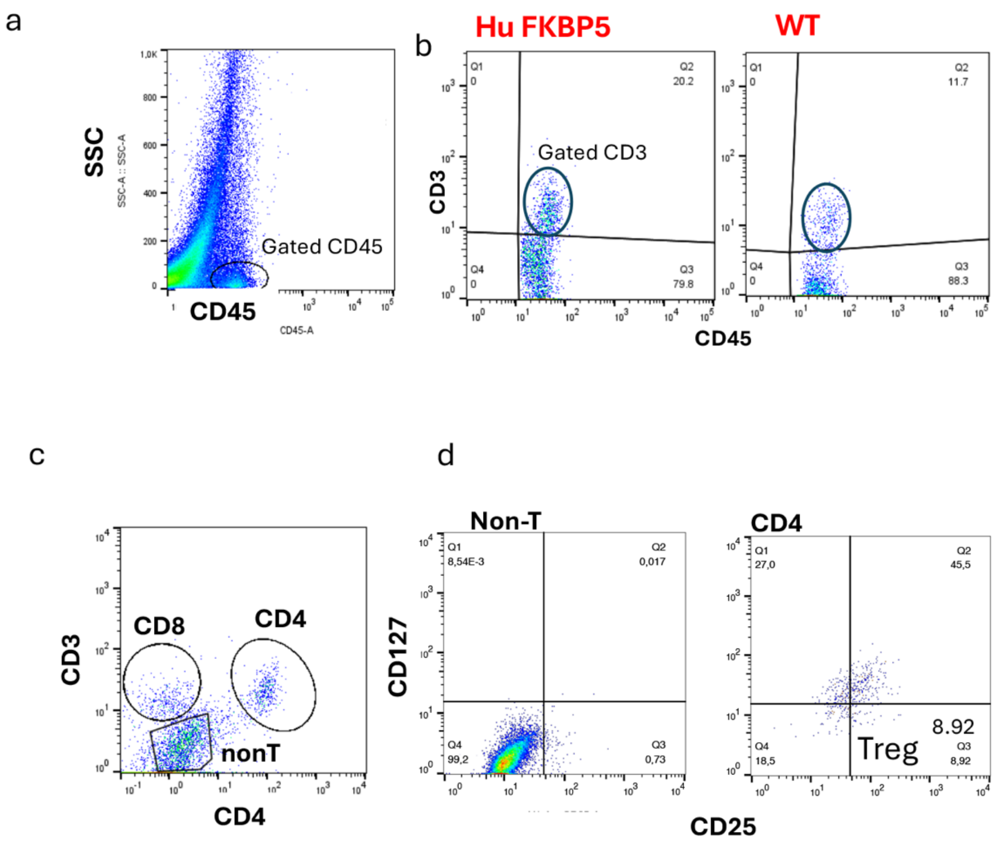

Fig S3

Figure S3. a, b Gating strategy used for the identification of CD3 T cells in the TME. Cells were first gated on CD45/SSC (a) to identify the leukocyte population. Subsequently, CD3 T cells were selected by gating on CD3/CD45 cells (b; left, huFKBP5 Tils; right, WT Tils). c, d Gating strategy used for the identification of Tregs in the tumor microenvironment. Cells were first gated on CD45/SSC (a). Subsequently, CD4 T cells were selected by gating on CD3/CD4 cells (c). Within the CD3/CD4 gate, regulatory T cells were identified based on CD127 and CD25 expression (d), with quadrant axes set according to the CD3-negative (non-T) in the CD127/CD25 dot plot.

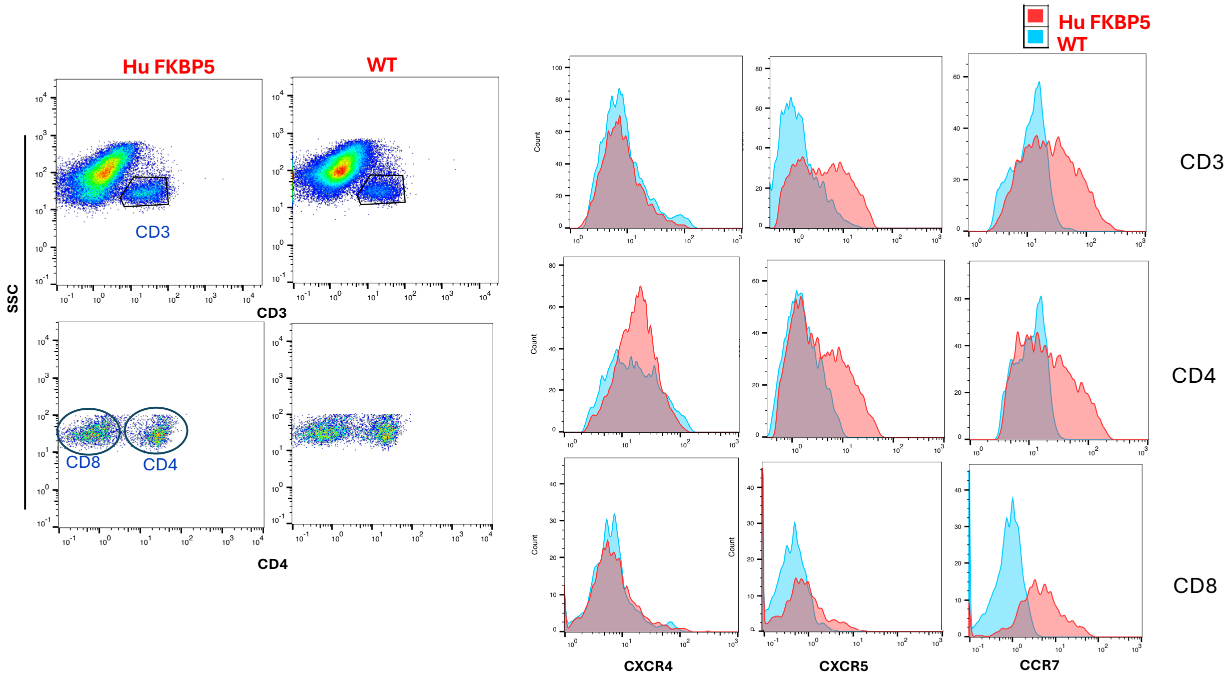

Figure S4 Gating strategy (left) and overlay histograms (WT, blue; vs huFKBP5, red) of expression of chemokines (right).

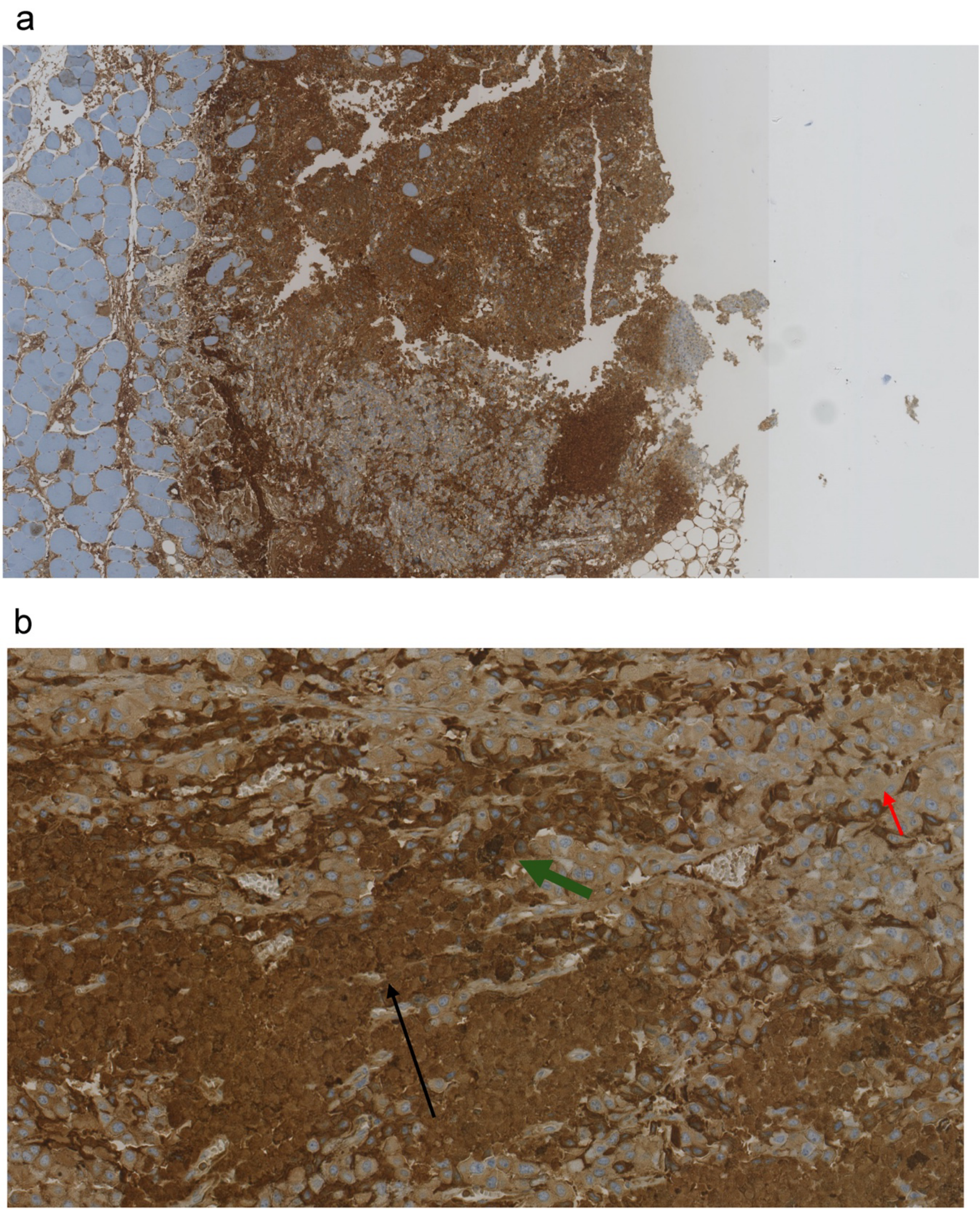

Fig. S5

Figure S5. Cleaved caspase-3 immunoreactivity: overviews of the tissue section in Fig 6b, left.
a, Low magnification overview (×5magnification). Strong diffuse cytoplasmic positivity is observed in tumor tissue. Skeletal muscle, on the left of the tumor, serves as internal negative control.
b, Cleaved caspase-3 immunoreactivity in peri-necrotic region (x20 magnification). Tumor cells surrounding a necrotic area (black arrow) display variable expression, with strongly positive cells (green arrow) and moderately positive cells (red arrow), indicating a gradient of necropoptotic activation in the peri-necrotic microenvironment.

Fig.4b

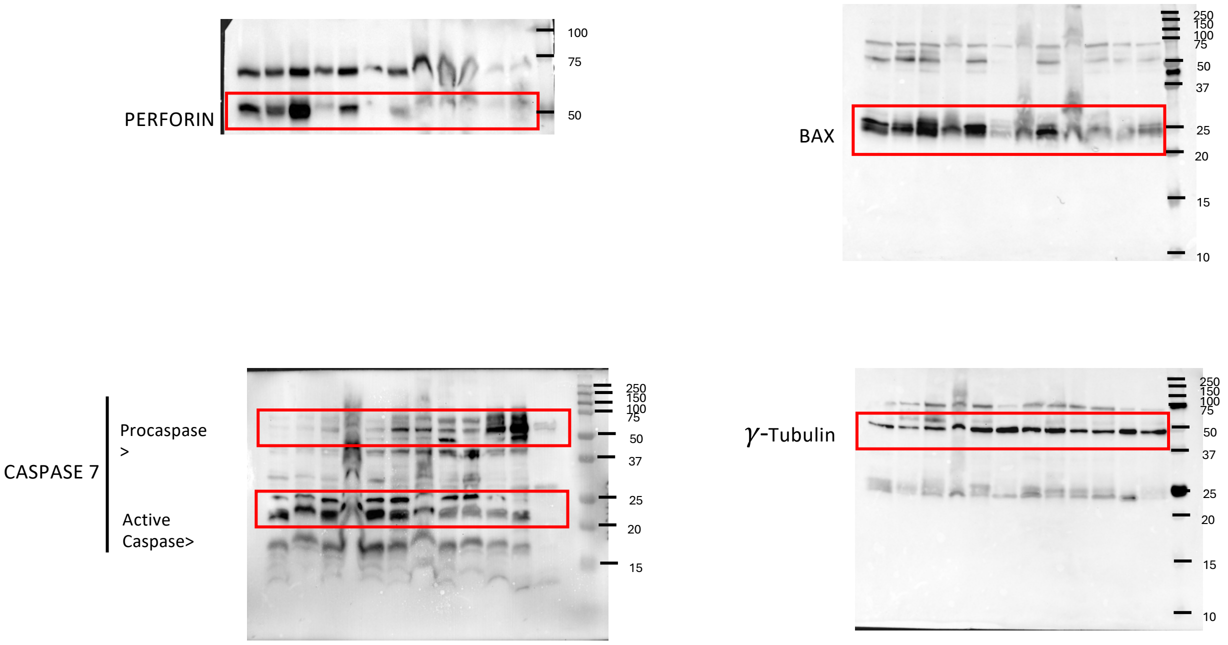
PERFORIN
100
75
50
BAX
250
150
100
75
50
37
25
20
15
10
CASPASE 7
Procaspase >
Active Caspase>
250
150
100
75
50
37
25
20
15
γ-Tubulin
250
150
100
75
50
37
25
20
15
10

Fig.4c

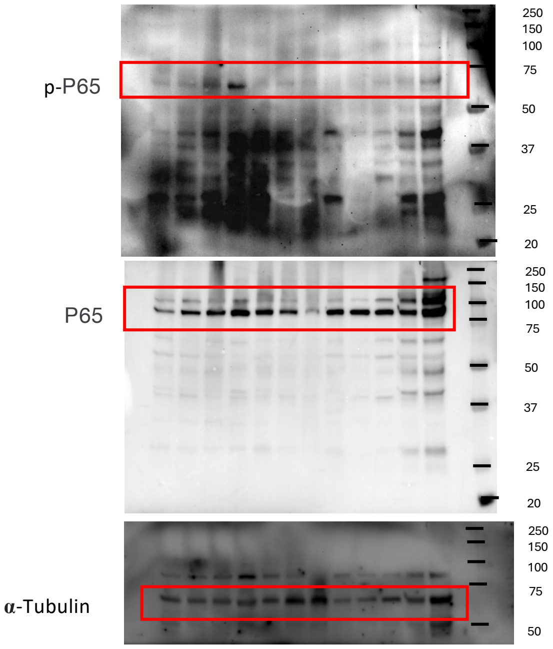
p-P65
250
150
100
75
50
37
25
20
P65
250
150
100
75
50
37
25
20
α-Tubulin
250
150
100
75
50

Fig.6c

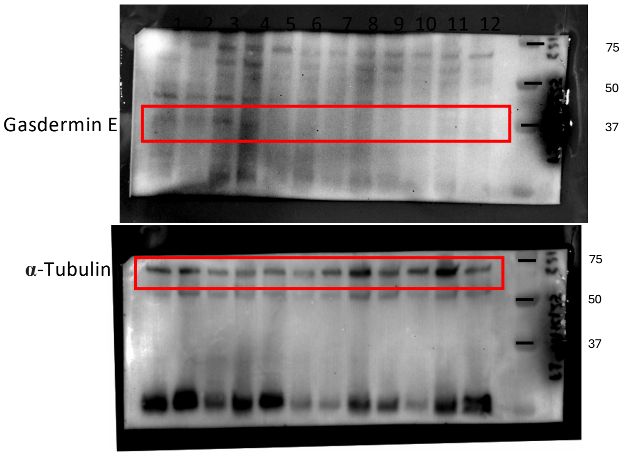

Supplement: Supplementary file 1 — Supplementary [file 41420_2026_3047_MOESM1_ESM.pdf]
